# Polyamine-Based Organo-Clays for Polluted Water Treatment: Effect of Polyamine Structure and Content

**DOI:** 10.3390/polym11050897

**Published:** 2019-05-16

**Authors:** Cinzia Cristiani, Elena Maria Iannicelli-Zubiani, Giovanni Dotelli, Elisabetta Finocchio, Paola Gallo Stampino, Maurizio Licchelli

**Affiliations:** 1 Politecnico di Milano, Dipartimento di Chimica, Materiali e Ingegneria Chimica “Giulio Natta,” Piazza Leonardo Da Vinci 32, 20133 Milano, Italy; elenamaria.iannicelli@polimi.it (E.M.I.-Z.); giovanni.dotelli@polimi.it (G.D.); paola.gallo@polimi.it (P.G.S.); 2 Università di Genova, Dipartimento di Ingegneria Civile, Chimica e Ambientale, Via all’Opera Pia 15, 16145 Genova, Italy; 3 Università degli Studi di Pavia, Dipartimento di Chimica, Via Taramelli 12, 27100 Pavia, Italy; maurizio.licchelli@unipv.it

**Keywords:** organo-clays, polyamines, clay-amine interaction mechanisms, structure effects, la uptake and release

## Abstract

Hybrid materials based on clays and polyamines are supposed to be efficient heavy metals sorbents due to the well-known adsorption behaviour of the clay matrix and to the coordination properties of un-protonated amino groups. For this purpose, a montmorillonite clay was modified with three different aliphatic polyamines: L6 and L10 have a linear structure with six and ten amino groups, respectively, while B14 is a branched polyamine with fourteen amino groups. Initial amine concentration was the main parameter investigated and data were fitted with Langmuir and Freundlich models. Interaction mechanisms between clay and amines were deeply investigated by different experimental techniques such as X-ray powder diffraction (XRD), thermal analysis measurements (DTG), Fourier Transform Infrared Spectroscopy (FT-IR) and diffuse reflectance (NIR) spectroscopy. Experimental results showed that the amount of amines efficiently immobilized in the solid phase can be increased by increasing the initial concentration of polyamines in the clay modification process. These data were best fitted by Freundlich model, indicating a presence of surface sites of different nature. In the resulting hybrid materials, neither the accessibility of the NH/NH_2_ groups of the amines, nor the accessibility of the structural OH of the clay was hindered. Several preliminary tests in La ions’ uptake and release from aqueous solution were also carried out. In the conditions used for this study, total metal ion removal was achieved at sufficiently low linear amine loadings (i.e., 0.45 mmol/g_clay_ for the small L6 amine), suggesting that these hybrid materials are promising for the proposed application in environmental remediation.

## 1. Introduction

In recent years, water issues have progressively gained prominence in view of water relationship with the three pillars of a sustainable development: that is, economic, social and environmental [1]. It is an undeniable fact that unsustainable development pathways have generated immense pressures on water resources, affecting its quality and availability and the Earth’s capacity to sustain the growing demands for freshwater is being challenged [2,3]. Water, indeed, is also a key resource for industrial and manufacturing processes (e.g., heating, cooling, cleaning, rinsing, etc.) but wastewater that is generated can cause environmental damage when it is discharged without any treatment. Industrial pollutants in water generally include heavy metals and /or huge amounts of organic pollutants whose removal is mandatory indeed but requires the application of complex and expensive processes [4]. The complexity and the cost of the treatments are much more pressing in case of very strict disposal limits, low concentration of the contaminants and/or contaminants hard to be removed.

Conventional techniques proposed for this purpose, such as ion exchange, precipitation, electrodialysis, reverse osmosis, ultrafiltration, flocculation, biosorption, adsorption and so forth [5,6] in some cases are not effective to treat complex and complicated polluted wastewaters or they are too expensive. For instance, conventional water treatment processes are not able to adequately address the removal of a wide spectrum of heavy metals (e.g., Cd, Cr, Cu, Ni, As, Pb and Zn), in particular, when they are present in complex mixtures [7].

Numerous approaches have been explored to develop cheaper and more effective technologies. Among these purification methods, the adsorption process, using adequate sorbents, is considered one of the most efficient and economical techniques in view of its simple design and facile handling [8] and the search for low-cost adsorbents with strong metal-binding capacities has been pursued. Consequently, sorption, particularly Solid Phase Extraction (SPE), has become one of the most promising alternative treatments technologies, being simple and potentially low cost. Many different solid adsorbents, such as materials of mineral, organic or biological origin, zeolites, industrial by-products, agricultural wastes, biomass and polymeric materials have been proposed and studied in the literature [9].

In this respect, the use of natural materials as sorbents has gained significant interest in recent years, mainly due to their high selectivity for certain heavy metal ions, their low cost and potential to be more environmentally friendly [10,11,12]. “Green adsorbents,” however, are expected to possess lower adsorption capacity than the super-adsorbents reviewed in the literature, thus a strong improvement of their properties is required to make them competitive. In the above “scenario,” clays have been suggested as a “green” alternative being characterized by low cost, high mechanical intensity, good tolerance towards harsh chemical environment, convenient solid-liquid separation and excellent reusability [13,14]. More recently, the use of natural clays in treating rare earths-containing wastewaters has been reported [15]. Montmorillonites were selected as sorbent in view of their excellent properties, including high cation exchange capacity, swelling behaviour, adsorption properties and large surface areas [15,16]. These clays, however, cannot efficiently remove huge amounts of pollutants and show a negligible selectivity towards the different metal ions. Therefore, layered inorganic-organic hybrid materials (organo-clays) have been proposed in various applications [17,18,19]. Indeed, montmorillonites can be easily modified by some intercalating agents, which can be allocated in the clay interlayer via a combination of ionic and weaker forces [20,21]. Some Authors, [22], with the final target of Rare Earths (RE) recovery, demonstrated that the use of organo-clays has positive effects on the capture of La ions. They investigated the intercalation of two different montmorillonites with *N*-(methoxy-polyethylene glycol) ethylene diamine. Such a polymer was selected for being characterized by a polyethylene-glycol chain, able to intercalate in the clay interlayer [23] and by two amino groups, already reported in the literature, able to interact and remove heavy metal ions [24,25]. Preparation procedure was deeply investigated at different polyamine concentrations and operating conditions [26]. It was found that the intercalation mechanism was mainly dependent on the pH of the preparation procedure, which affects the protonation of the amino groups. At alkaline pH, the interaction was mainly based on weak bonds between the free amino groups of the organic moiety and the clay matrix and the obtained organo-clays were much more effective in La uptake than pristine clay. So, as general remark, the clay-polymer system can be considered promising for the here proposed application.

Starting from these data, in the present paper new organo-clay systems are assessed where montmorillonite is modified with polyamines characterized by longer chain length (i.e., larger number of amino groups) or more complex structures (i.e., more steric hindrance). By the use of multi-technique characterization approach (i.e., X-ray powder diffraction, XRD, thermal analyses measurements, TGA-DTG, Fourier Transform Infrared Spectroscopy, FT-IR and Diffuse Reflectance Near Infrared Spectroscopy, DR-NIR) the interaction mechanisms occurring between the clay matrix and the polyamines are proposed. The final goal is to identify the best operating conditions, with respect to the chemical nature of the polyamine, to prepare organo-clay system characterized by high metal capture capability and selectivity in wastewaters treatment. Finally, the prepared materials were tested in La ions uptake to assess their usability in the field of environmental water remediation.

## 2. Materials and Methods

### 2.1. Materials

A natural smectite clay was used as sorbent, namely a Ca-montmorillonite (STx-1b, The Clay Minerals Society, STx hereafter). From the supplier datasheet, the chemical composition of the clay is ^IV^Si_4.0_^VI^(Al_1.21_Fe^3+^_0.05_Mg^2+^_0.36_Ti_0.02_)^XII^(Ca_0.14_Na_0.02_K_0.01_)O_10_(OH)_2_ and the measured Cation Exchange Capacity (CEC) is 89 meq/100 g.

Three different polyamines were used as clay modifiers. Polyamines are characterized by several amino group numbers and/or more hindered structures. In particular, two linear ethylene-based amines with different chain length (L6 and L10) and a branched amine (B14) were considered (Table 1, where chemical structures and labels used within the text are summarized).

Polyamine L6 technical grade was supplied by Sigma Aldrich; L10 and B14 were prepared according to literature procedure [27].

### 2.2. Organo-Clays Preparation

The preparation of the organo-clays was performed according to a procedure developed elsewhere [28], which implied the mixing of the clay with the aqueous polyamine solution, in a jacketed reactor under vigorous stirring (500 rpm), for 90 min at controlled temperature of 30 °C. This temperature and time were demonstrated to be the proper ones for the effective amine immobilization, while amine concentration was the main parameter to be considered to affect the intercalation process [23].

The pH of the solutions was measured before and after the reaction using a Mettler Toledo FE20/EL20 digital pH-meter (Mettler Toledo, Italy). All the polyamine solutions were highly basic, presenting a natural pH of about 11 and no variation of this parameter was detected during the mixing reaction.

At the end of the reaction, the solid and liquid phases were separated by centrifugation (3500 rpm for 15 min, RotoFix 32 centrifuge HETTICH, Tuttlingen, Germany). The dried solid (one day at r.t.), was ground in a mortar and fully characterized.

These measurements could be affected by underestimation due to the entrapment in the interlayer of polymers combustion residues.

According to the literature, quantitative evaluation of the amine contents was performed by Chemical Oxygen Demand (COD) [23,28] using a Spectrodirect Lovibond instrument (Tintometer Gmbh, Dortmund, Germany). Measurements were performed according to Reference [29]. Amine content in the solid phase (*C*_s_) was calculated by difference between the initial (*C*_0_ mmol/g) and the residual polyamine amount in the liquid phase (*C*_res_ mmol/g) according to Equation (1):

(1) $$C_{s}=C_{0}-C_{\mathrm{res}}$$

After the interlayer reaction, also the presence of Ca, that is, the clay interlayer cation and Mg, leached from clay layers or present as impurity, was monitored in the solution. Ca and Mg were measured by Inductively Coupled Plasma—Optical Emission Spectroscopy (ICP‒OES) analyses using a Perkin Elmer Optima 2000DV spectrometer, estimated error within 1%).

Throughout the text, all the results of chemical analysis will be referred to 1g of clay.

In Table 2, labels of the samples, initial amine solution concentration (mM) and *C*_0_—initial mmoles of polyamine per g of clay—are summarized.

### 2.3. Organo-Clays Characterizations

X-ray powder diffraction (XRD) patterns were recorded with a Bruker D8 Advance diffractometer using a graphite-monochromated Cu Kα radiation; the scan step was 0.02° 2θ and the measurement time was 1 s per step. The XRD line profile analysis was performed with TOPAS P 2.1 software (Bruker AXS, Karlsruhe, Germany) using a Pearson VII profile function, after background subtraction. The calculated profiles were used for the determination of basal spacing (d_001_) of the organo-clays.

Thermal analyses measurements (TG-DTG) were performed with a DTA-TG SEIKO 6300 thermal analyser. The experiments were carried out in flowing air in the temperature range of 25–1000 °C, with heating rate of 10 °C/min.

FT-IR spectra were recorded with a Jasco mod. 615 spectrometer and with a FT-IR Thermo Nicolet 380 Avatar (Thermo Electron Corporation, Madison, WI, USA) using the KBr pressed disk technique (mid-IR region).

Diffuse reflectance NIR spectra of pure powders have been obtained in air by a Jasco V570 UV-vis-NIR instrument (Jasco-Europe SRL, Jasco^TM^ Software) and reported in Kubelka-Munk units in the range 9000–4000 cm^−1^.

### 2.4. Metal Ions Uptake and Release Tests

La(NO_3_)_3_ solutions were selected as model systems. La ions uptake and release experiments were performed according to literature [15,26,28]. The use of concentrated solutions was considered in order to demonstrate the feasibility of the method in industrial applications.

Briefly, weighted amounts of organo-clays were contacted under stirring with model solutions 19 mM concentration for 90 min, solid/liquid ratio = 0.04 g/mL. pH of the contacting solution was in the range 5–6, that is, pH of the starting La solution before contacting. Solid and liquid were separated by centrifugation (HETTICH 32 RotoFix).

**Figure 1:**
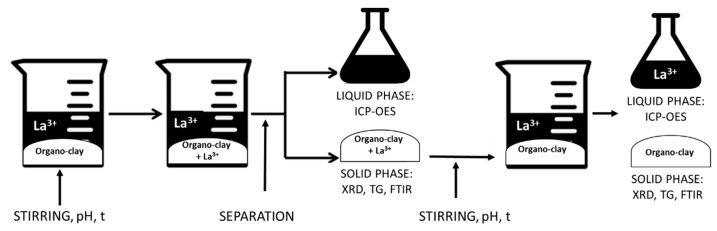
Flowchart of the UPTAKE/RELEASE process.

The flowchart of the uptake/release procedure is reported in Figure 1.

La ions content in the solutions, (mother, after uptake and after release), was determined by ICP-OES (Perkin Elmer Optima 2000DV spectrometer).

Captured ions were determined according to Equation (2):

(2) $$q_{t}=\frac{\left(C_{0}-C_{t}\right)V}{W}$$

where *q_t_* (mmol/g) is the adsorbed metal ion at time *t*, *C*_0_ and *C_t_* are the initial and the residual metal concentrations in solution (mmol/L) respectively, *V* is the volume of solution (L) and *W* is the organo-clay mass (g).

Release tests were performed by contacting the La-containing organo-clays with HNO_3_ solution at pH 1 under continuous stirring at room temperature, for 1.5 h, with a solid/liquid ratio of 0.026 g/mL [30].

Released ions *q_w_* (mmol/g) were calculated according to Equation (3), where *C_w_* is the metal concentration in solution (mmol/L) after release (determined by ICP-OES).

(3) $$q_{w}=\frac{\left(C_{w}\right)V}{W}$$

*V* is the volume of the solution (L) and *W* is the organo-clay mass (g).

## 3. Results and Discussion

### 3.1. Characterization of Modified Clays

**Figure 2:**
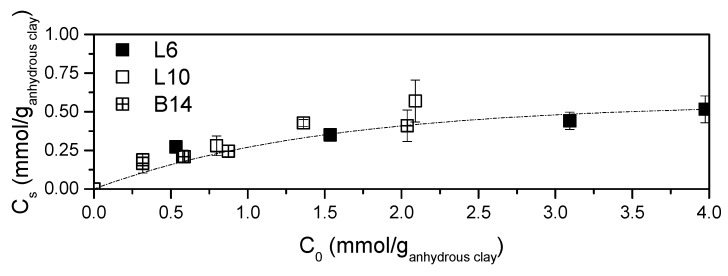
Amine content in the solids as a function of the initial contacted amine.

In Figure 2 polyamine contents (*C*_s_) obtained at different initial amine concentrations are reported. The extent of the intercalation reaction is clearly irrespective of amine structure and molecular weight. The asymptotic behaviour of Figure 2 well fits all the data. The plateau value is reached, by all the systems, for *C*_0_ higher than 0.3 mmol/g_anhydrous clay_ and this value represents the maximum loadable polyamine in STx clay in these conditions.

Considering that the intercalation reaction was performed at pH = 11, that is, pH of the polymer solutions, it can be assumed that polyamines interact with the clay in their neutral form and no quaternary ammonium ions should be present in the organo-clays.

Modified-clays were fully characterized to get information on the polymer-clay interaction.

**Figure 3:**
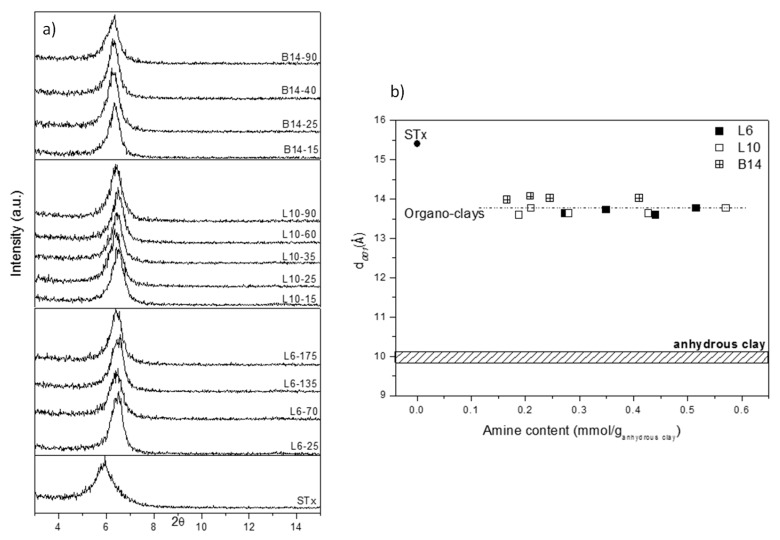
(**a**) X-ray diffraction (XRD) analysis of pristine and modified clays prepared at different initial amine concentrations and (**b**) Basal spacing (d_001_) as a function of the amines content of modified clays, (for sake of comparison, d_001_ of fully hydrated and dehydrated STx are reported).

X-Ray diffractograms of the samples are reported in Figure 3a, where pristine clay (STx) is also reported for comparison.

A displacement of the basal reflection from the original STx towards higher angles is evident, which corresponds to a contraction of the interlayer spacing d_001_ from 15.4 to 13.5–14 Å for all the organo-clays, regardless the amines structure and content.

To better clarify this point, in Figure 3b d_001_ are plotted as a function of the corresponding amine content. For sake of comparison, also pristine STx and pristine STx fully dehydrated are also reported. It is evident that all the d_001_ of the modified-clays are very close and their values are intermediate between pristine-fully hydrated and pristine-fully dehydrated clays.

The observed interlayer shrinkage, calculated from XRD, is an indication of the presence of the polyamine interacting with clay interlayer [31]; indeed, no other effect than polymer-clay interaction should be present in this case. However, usually, the accommodation of large organic molecules, such as polyethylenglycols PEG [23,32], inside the clay has been reported to cause interlayer enlargement [33,34,35]. A contraction of the interlayer spacing has been reported in the literature for the intercalation, via ion exchange mechanism, of a polycationic quaternary amine polymer [31]. Nevertheless, in the considered samples, no cation exchange is expected: intercalation reaction was performed at the amine original pH of 11, that is, basic enough to prevent any protonation of the amino-groups or any protonation of the clay interlayers. To verify this point, ICP analyses were performed on the solution after the polyamine-clay contacting reaction: in case of cations exchange the presence of interlayer ions, for example, Ca^2+^, has to be found. As expected, no Ca^2+^ ions were found after intercalation, thus confirming that no ions exchange has occurred, and amines are intercalated in their neutral form.

A possible explanation for the observed interlayer shrinkage could be found by considering interlayer water molecules. As reported elsewhere for PEG-based systems, the interaction between the intercalated polymers and the clay involves the formation of weak bonds between the polymer chain and the interlayer water molecules that are in turn coordinated to the interlayer cations [23,31,32]. Similar effects can be claimed also in this case. The interlayer contraction, indeed, could imply a water-amine interaction that, in view of the higher affinity of the amino groups for the interlayer cations could result in water displacement. Moreover, interlayer spacing calculated for the organo-clays, are intermediate between fully hydrated- and fully dehydrated-pristine clay, thus supporting this explanation. Thus, the polyamine chain, which possesses a stronger coordination capability towards the interlayer cations, could displace some interlayer water molecules. The replacement of part of the interlayer water molecules with these new “stronger and shorter” coordination bonds could result in the observed contraction. However, a minimum interlayer water content cannot be overcome to prevent either repulsion phenomena between two faced silico-aluminate layers or the structure collapse. This phenomenon can account for the observed (d_001_) constancy in all the modified-clays.

On the contrary, in the case of PEG intercalation [23,32], in view of the polyoxyethylene chains of the polymer, a weaker H-bond interaction between the etheric oxygen of the polymer chain and the interlayer water molecules occurred. Indeed, water displacement was hardly detected and the hindrance effect of the PEG chain prevailed, resulting in an interlayer enlargement.

To confirm this picture, TG analyses of the solids were performed in air: both polyamine decomposition and the water evolution are detectable by this technique.

**Figure 4:**
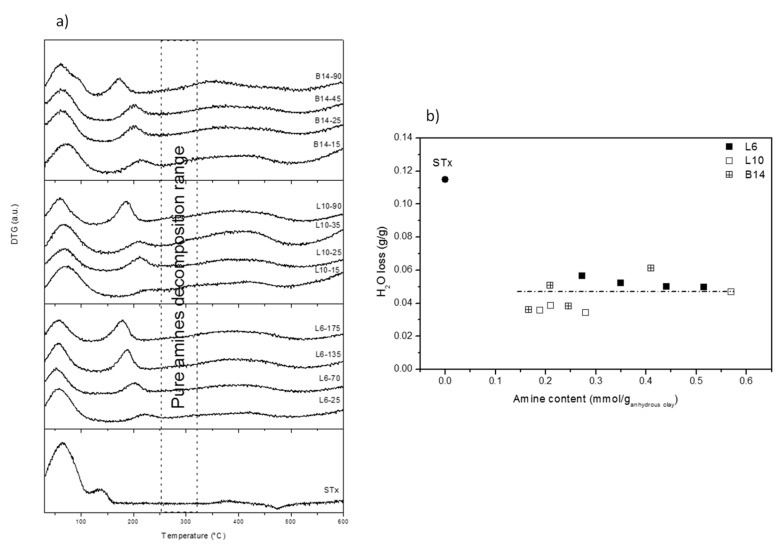
(**a**) DTG curves of pristine and modified-clays prepared at different polyamine content; and (**b**) Water loss (calculated from thermal decomposition) as a function of the amine content for pristine and modified clays.

DTG curves are shown in Figure 4a. Once more, very similar behaviours were observed for all the different samples.

Three different ranges of temperature can be identified: (1) 100–120 °C corresponding to water evolution; (2) 150–250 °C corresponding to amine decomposition; (3) 300–450 °C corresponding to decomposition of combustion residue and clay collapse.

The above thermal phenomena are discussed below in details. The thermal phenomenon up to 100–120 °C is present in both pristine and all the modified clays. It accounts for water evolution. However, broader decomposition patterns, accompanied in some case by shoulders, are found in organo-clays with respect to the pristine one. In case of modified-clays, water evolution corresponds to sample water content, mainly interlayer water molecules. Indeed, organo-clays after intercalation were dried at room temperature for one day, conditions that are too mild to modify the interlayer water content that is, therefore, preserved. However, as discussed above, “different” interlayer water molecules are present in the materials, some of them interacting with the clay interlayer cations and some others interacting with the polyamine chain. These last water molecules, in view of their stronger interactions, evolve at temperatures that are slightly different from the others. Therefore, this heterogeneity of bond strength can explain broadness and complexity of the first thermal decomposition.

Water losses were quantified and plotted as a function of the total intercalated polyamine content (Figure 4b). Very similar water losses were found for all the samples, irrespective of the polyamine content or structure and definitely lower if compared to the unmodified clay.

This behaviour is in line with d_001_ reduction (compared to the unmodified STx) and constancy (for all the modified clays) and confirms that XRD findings were related to interaction of interlayer water and intercalated polyamine. Moreover, it also strongly supports the presence of the threshold amount of water that has to be retained to preserve the clay structure.

Thermal phenomenon at 150–250 °C, not detected in the pristine clay, is attributed to the intercalated polyamines decomposition, which in pure amines are observed at about 300 °C. With respect to the free amines, in the organo-clays the decomposition is anticipated of about 100 °C. In addition, a progressive decrease of the decomposition temperature was observed on increasing amine content in the solid. In line with literature indication, anticipation of polymer decomposition is attributed to the effect of the polymer-clay interaction [23].

Moreover, the last thermal phenomenon, detected between 300 and 450 °C, also in this case is possibly related to decomposition of pitch-like residues of combustion of the polyamine [23]. Such residues trapped inside the clay thus require higher temperature to be decomposed. [23,32].

IR spectra of the L6-70 material as pure powder were also recorded at increasing temperatures from room temperature to 400 °C in air (Supplementary Material, Figure S1). Pure powder spectra show very strong and noisy bands due to the high concentration of the organic loading. Nevertheless, two broad and ill-defined absorptions are detected, characterizing the amine molecules and corresponding to CH stretching modes (3000–2800 cm^−1^) and NH stretching modes (3300–3200 cm^−1^) as detailed in the following. Their intensity strongly decreases upon heating up to 200 °C and above, as further confirmation that organic moieties are mainly responsible of the weight loss detected in this temperature range by TG studies.

**Figure 5:**
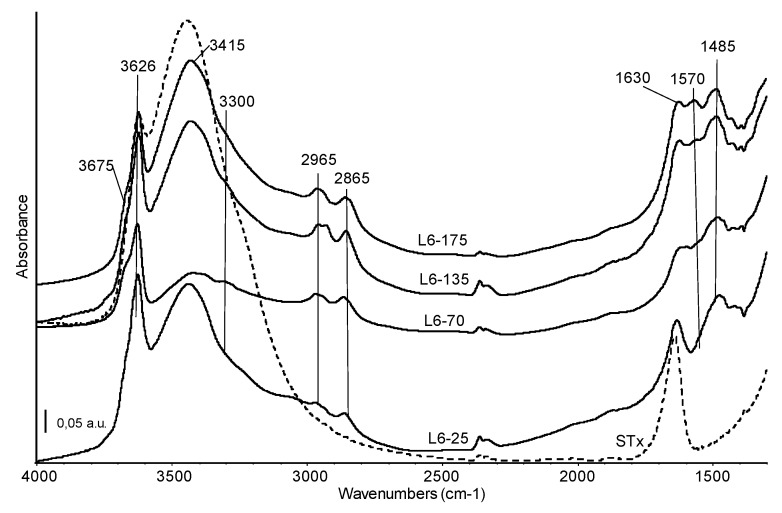
FT-IR skeletal spectra of the organo-clays prepared at different initial L6 amine content (common scale). Broken line: FT IR spectrum of the reference STx material.

Therefore, from XRD and DTG combined information, it is possible to state that all the polyamines show quite similar interactions with the clay, partially due to intercalation by means of weak bonds and partially due to water interactions, suggesting the presence of a multilayer and heterogeneous situation. Despite a good accordance among XRD and TG experimental data, the presence of some polyamine adsorbed at the surface clay platelets cannot be excluded. Therefore, confirmation of the supposed mechanisms was also assessed by skeletal IR spectra. For this purpose, organo-clay containing L6 was selected. Indeed, the L6 structure is simpler than the other polyamines and, as a consequence, data interpretation is expected to be simpler too. Moreover, it is a commercial product, therefore much more suitable for the production of the organo-clays for industrial application. Organo-clays containing increasing amount of L6 polyamine were hence analysed and resulting spectra are reported in Figure 5. Spectrum of the pristine STx material (dashed line) is reported for sake of comparison.

Skeletal bands of the pristine montmorillonite material are not affected by the modification with amine and the corresponding spectral region (below 1300 cm^−1^) has not been reported in Figure 5.

The high frequency region of all the spectra shows bands assigned to OH stretching modes at 3626 cm^−1^ (structural OH groups of montmorillonite, mainly coordinating central Al atoms) and 3415 cm^−1^ (water molecules), overlapping the broad band due to water vapour adsorbed on the KBr matrix [32,36]. The introduction of amines leads to changes in shape and relative intensity of these features. In particular, in this region a decrease in intensity of the band due to water molecule can be detected, especially for the L6-70 sample. The ratio water molecules/structural OH is always slightly lower in the organo-clays than in the pristine STx spectrum and suggests the preferential interaction of amine with water, either adsorbed water either interlayer water, which is partially substituted by the organic molecules. This effect could be considered indirect evidence of some amine intercalation. A weak absorption centred at 3300 cm^−1^, more evident in the L6-70 spectrum, is assigned to the NH stretching mode of a secondary amino group. Two absorptions, quite complex, at 2965 and 2865 cm^−1^ correspond to the CH asymmetric and symmetric stretching modes of the CH_2_ of the alkyl chain. The complexity of these bands, which are evidently split in the spectrum of the L6-135 sample, is due to the existence of CH groups having different environment, that is, in the –(CH_2_)_2_– unit or linked to –NH– groups. Broadening and splitting of these bands is indeed related to the interaction of the chain with the clay [37]. At lower frequencies, the main bands corresponding to the amine chain are detected at 1570 cm^−1^, NH deformation mode and at 1480, 1430 and 1390 cm^−1^, CH deformation modes. The relative intensity of the amine band at 1570 cm^−1^ increases with the increasing amine content in the starting solution, confirming the efficiency of the preparation procedure. Moreover, the detection of this band highlights that amino groups in the organic framework are still “free” after interaction with the clay and are therefore available for the uptake of metal ions. As expected, considering the highly basic contacting pH, no amine protonation was detected.

In sum, the immobilization of the organic chain in solid matrix should not hinder the accessibility of the NH/NH_2_ groups, which are directly involved in the coordination of metals in environmental remediation applications [24,38].

**Figure 6:**
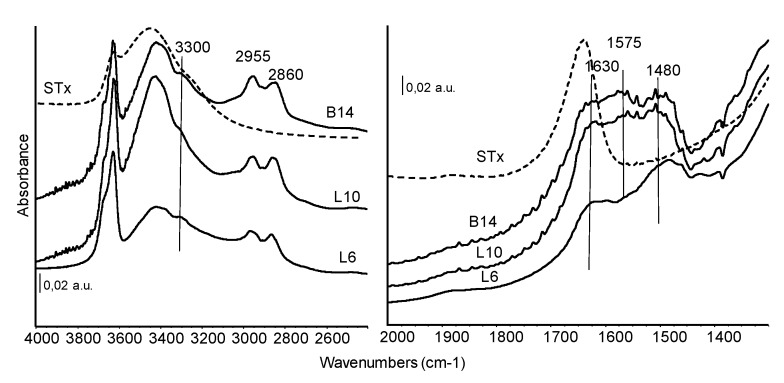
FT-IR skeletal spectra of all the organo-clays samples prepared at similar initial amines molarity. Dashed line: STx reference spectrum.

FT-IR spectra of organo-clays prepared at similar initial amine concentration but with different polyamines are shown in Figure 6 and exploited in the regions of interest.

As discussed in the previous paragraph, in the high frequency region of the spectra, the band centred at 3300 cm^−1^ ca. is assigned to NH stretching of the secondary amino groups. The –NH_2_ vibrational stretching modes fall in the same region, while the corresponding asymmetric stretching absorptions could be masked by the band of H-bound water centred at 3350 cm^−1^.

Bands at 2955 and 2860 cm^−1^, are characteristic of the vibrations of CH bonds in CH_2_ groups. Correspondingly, the asymmetric CH deformation mode is detected at about 1480 cm^−1^ ca quite broad and complex. In this spectral region, also other contributions can actually be identified, although strongly overlapped. The absorptions in the range 1635–1620 cm^−1^, shifted in wavenumber in comparison to the pristine material, are assigned to deformation modes of water. Another broad absorption growing at 1575 cm^−1^ is due to NH deformation mode, slightly stronger in L10 and B14 sample.

The comparison of Figure 5 and Figure 6 showed similar spectral features for the different organo-clays: NH stretching and deformation modes of exposed amino groups, reduced water content of these materials in comparison to the pristine clay, complex CH stretching and deformation modes slightly perturbed by the interaction with the matrix. As already reported for XRD and TG the presence of only very few and small differences, suggest that similar immobilization mechanisms occurs, in spite of different structure or molecular weight of the considered polyamines.

**Figure 7:**
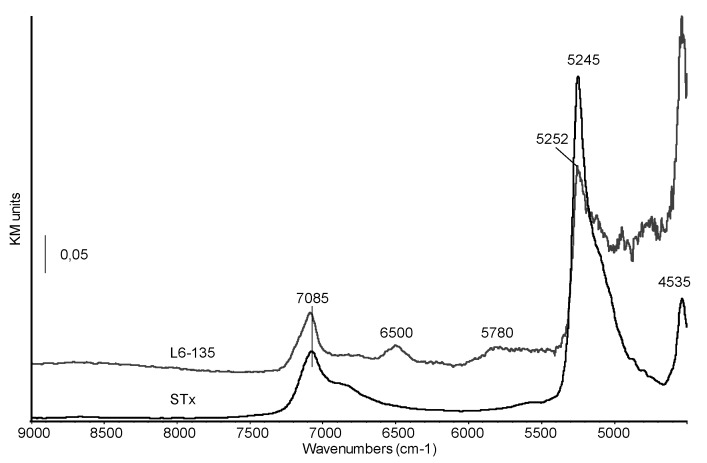
Diffuse Reflectance NIR spectra of STx and organo-clay L6-135.

Diffuse Reflectance-NIR spectra of pristine montmorillonite and of sample L6-135 are reported in Figure 7.

In the pristine clay spectrum, the complex band at 7085 cm^−1^, with a shoulder at 6865 cm^−1^, corresponds to the overlapping of the first overtone of vibrational modes of structural OH stretching and water molecules and to the first overtone of vibrational modes of water molecules strongly bonded through H-bonds. The sharp band centred at 5245 cm^−1^ and tailing towards lower frequencies has been previously assigned to a (ν+δ) combination mode of water strongly interacting with interlayer cations and with the clay surface [39]. The NIR spectrum of the organo-clay shows a significant decrease in the relative intensity of this band which is also shifted at higher frequencies (5252 cm^−1^). The modification of this feature suggests that in the studied organoclays the immobilization process affects interlayer water, as also reported by some of us for PEG intercalation in a previous paper [40].

On the other side, the shift in wavenumbers suggests that the strength of the H-bonds between residual water molecules is also changed, possibly increased [37,41]. The band at 4535 cm^−1^ is assigned to overtones of structural OH groups which seem to be only marginally involved in the amine immobilization. Finally, new features appear at 6500 and 5780 cm^−1^, corresponding to overtones and combination vibrational modes of –NH– and –CH_2_– groups, respectively, thus characterizing the organic chain.

To model the polyamine-clay interaction and trying to support the hypothesis of a complex and heterogeneous situation, the experimental results concerning the polyamines adsorption were interpreted according to Langmuir and Freundlich models [42,43]. It is well known that Langmuir model describes a more homogeneous situation where equal active sites can be occupied only once. On the contrary, Freundlich model is able to describe complex and heterogeneous (multilayer) surfaces.

Langmuir model parameters were calculated according to Equation (4):

(4) $$\frac{c_{e}}{q_{e}}=\frac{c_{e}}{q_{\max}}+\frac{1}{q_{\max}K_{L}}$$

where *q*_e_ (mg/g) is the adsorbed amount at equilibrium, *C*_e_ (mg/L) is the equilibrium concentration, *q*_max_ is the maximum monolayer adsorption capacity of the adsorbent and *K*_L_ is the Langmuir constant related to the free energy of adsorption.

Freundlich model parameters were obtained according to Equation (5):

(5) $$\log_{10}q_{e}=\log_{10}K_{F}+\frac{1}{n}\cdot \log_{10}C_{e}$$

where *q*_e_ and *C*_e_ have the same meaning of Equation (4), while *K_F_* ((mmol/(g·(mmol/L)^1/*n*^)) and 1/*n* are Freundlich constants related to the adsorption capacity and the adsorption intensity, respectively.

Calculated values of Langmuir and Freundlich constants are compared in Table 3.

**Figure 8:**
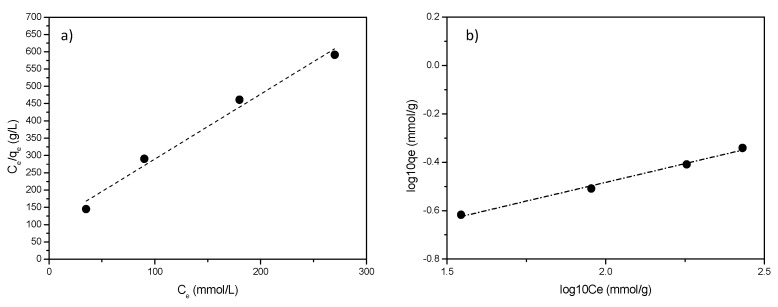
Adsorption isotherms for L6-STx: (**a**) Langmuir and (**b**) Freundlich model.

Plots of the linearized isotherms evaluated using both models are reported in Figure 8.

Only small differences were found (Figure 8a,b), however, despite data are only slightly better fitted, Freundlich model is selected as the most proper one to account for the adsorption. This is not surprising, considering that Freundlich empirical model can be applied to multilayer adsorption, with non-uniform distribution of adsorption heat and affinities over sites of different nature, as is the case of the clay here discussed [44]. Freundlich slope, ranging between 0 and 1, is a measure of adsorption intensity or surface heterogeneity: a larger heterogeneity of surface sites is present as the slope value is near to zero. Values of *n* > 1 indicate either penetration of a sorbate into a sorbent structure or lateral sorbate-sorbate interactions. In the case here reported, for all the considered polyamines, *n* > 1 indicates most probably lateral interactions between polyamine molecules in an adsorbed state [45].

### 3.2. Metal Uptake and Release

**Figure 9:**
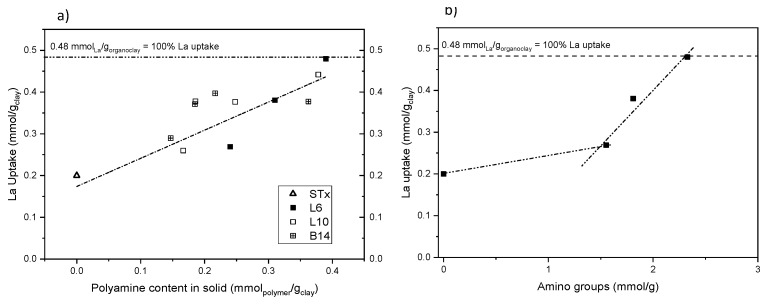
- La uptake as a function of (**a**) the polyamine content for the different organo-clays and (**b**) number of amino-groups (STx-L6 and STx-L10). Dashed lines are drawn as guide for the eyes.

Finally, the effect of the nature and the concentration of polyamine on metal capture reaction was performed. A parallel study was performed on the 3 systems, that is, L6, L10 and B14. Uptake reaction at initial La concentration 19 mM (90 min contact time and pH about 6) was performed using different organo-clays prepared at different polyamine initial concentration (10–90 mM) Results are reported in Figure 9a.

All the capture data can be accounted for a linear La uptake up to polyamine content of 0.45 mmol/g_clay_. At that value, 100% of La ions’ uptake, that is, total metal ions removal from the solution, was achieved. In particular, the organo-clay functionalized with the smallest amine chain L6 shows good adsorption capacity, that is, 65 mg/g sorbent, fitting indeed within the adsorption results reported in the open literature for Lanthanum uptake, as very recently reviewed by Iftekhar et al. [46].

On average, the uptake reaction is apparently insensitive to the polyamine structure that is, linear or branched and high or low molecular weight, showing a similar linear La uptake. In case of B14-based organo-clay, the lower uptake at increasing amine content is probably due either to lower polyamine content inside the clay or lower availability of the metal coordination sites. Indeed, in view of the highly branched structure of B14, lower polyamine content can be allocated inside the clay and/or the branched chains could interact each other, preventing some coordinating sites. Therefore, this suggests that the capture mechanism could be driven by the number of coordination centres, that is, the number of amino groups in the functionalized clays.

This effect becomes evident when uptake data obtained for L6- and L10-based materials are plotted as a function of the overall number of amino groups, (calculated as the product of the moles of polyamine present in the organo-clay and the number of amino groups per moles of polymer) (Figure 9b). Behaviour of L6- and L10-based materials is so overlapped that the organo-clays becomes indistinguishable, despite of the marked differences between the polyamine structures.

This suggests that the same total amount of amino groups, although included in molecules having different chain length, either linear or branched, has the same metal uptake behaviour. Therefore, shorter and simpler polyamines, easier to handle—for instance, more soluble—can be used without lacking effectiveness in the uptake reaction.

Also release experiments were performed. Knowledge on release mechanism will be useful in case of recovery of the captured metals with their revalorization and re-use as final target. In addition, desorption data could provide additional information to better understand capture mechanism and its relationship with polyamine loading, structure and composition.

The La-enriched organo-clays were thus contacted with solutions at pH 1, value that was proved [47] to be the best to perform the desorption reaction.

**Figure 10:**
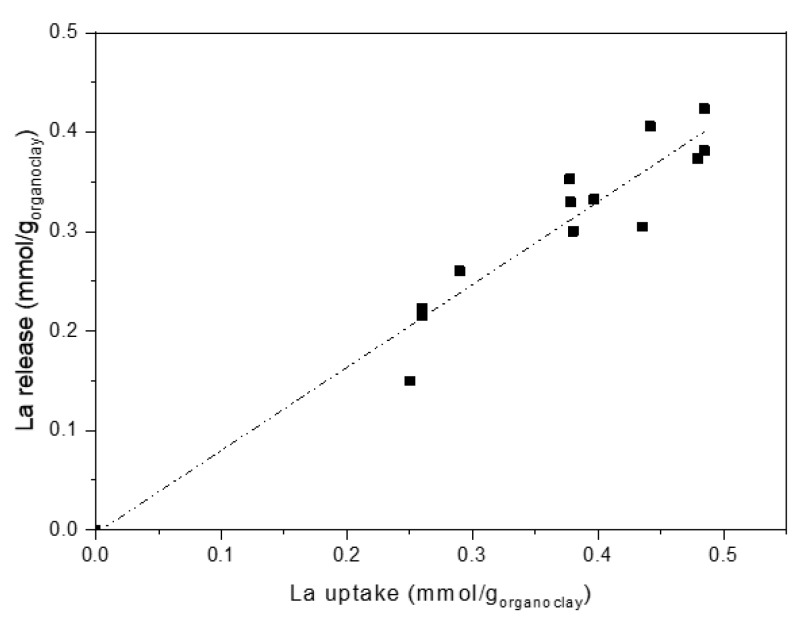
Lanthanum release as a function of lanthanum uptake (Dashed-dotted line: linear fitting).

Data obtained from release tests are reported in Figure 10, where La release is plotted as a function of La uptake. Also, in the case of release, very close behaviours were found for the different organo-clays. Once more, regardless of polyamine nature, release results can be fitted by a linear plot, where the different systems are indistinguishable.

This behaviour confirms that metal ions uptake is amino-groups dependent only. Indeed, considering release conditions, that is, pH = 1, La ions are recovered by replacing them with protons via acidification of the amino groups of the polyamines, whatever their structure. Amino-groups mainly interact with the ions (and not with the clay), therefore are free and easily protonable. The complete conversion of amine to ammonium results in an almost quantitative metal release (90–95%) that accounts for the linear plot (fitting line near to bisect) observed in Figure 10.

COD analysis of the solution after both uptake and release tests, evidenced the presence of very limited amount of polyamine, lower than 1% of the total content. Therefore, data reported on capture and release fit well with the hypothesis of significant polyamine immobilization in the interlayer.

Indeed, a conclusive picture of the clay-polymer interaction cannot be drawn on these bases and possibly a deeper and dedicated investigation by means of other techniques such as TEM and XPS is still required. Evidences from different characterization techniques, such as: a) a slight modification of the d_001_ observed by XRD; b) the anticipation of the polymer decomposition temperature, due to polymer-clay interaction as already observed by TG for similar systems; c) a decrease of water content due to interlayer water displacement detected both by TG and spectroscopic techniques; e) a negligible amount of polymer dissolved in harsh conditions (i.e., soaking and stirring at pH 6 and 1 for uptake and release test, respectively), accounted for polyamines molecules strongly interacting with the clay matrix. However, it cannot definitely discriminate between intercalated polyamine and/or polyamine strongly bonded to the platelets surface and edges.

## 4. Conclusions

The preparation of organo-clays using polyamines as modifying agents was carried out and the main following conclusions can be drawn:

1. amine content in the solid phase can be increased, increasing the initial amine concentration in solution up to a threshold value;

2. Freundlich isotherm model was found to be the one best fitting the data, meaning the presence of surface sites of different nature. This fact is not surprising because Freundlich isotherm is widely applied in heterogeneous systems especially for organic compounds [45].

3. Common mechanisms occur, in spite of different structures or molecular weights: at pH 11 polyamines are likely intercalated in the clay interlayer, although surface adsorption cannot be ruled out. Both interactions involve preferential interaction of amines with water, either interlayer water either adsorbed water (the first being at least partially substituted by the organic chains).

4. At lower amine content the accessibility of the amino groups of the polyamines is not prevented. Increased amine content leads to a significant interaction between the chains, somehow hindering the availability of the NH groups, namely for the branched chain. On the other side, amine linear structure does not seem to affect uptake activity. So, shorter and linear polyamines, more easily handled, can be used preferentially

5. In our conditions (19 mM initial La concentration, 90 min contact time and pH about 6), total La^3+^ removal was achieved already using the L6 hybrid material at quite low amine loading, that is, 0.45 mmol/g_clay_. An almost quantitative ions release was obtained by lowering pH of the aqueous medium. Thus, uptake and release results suggest that polyamine-based organo-clays are promising materials for the proposed application.

## Figures and Tables

**Table 1:** Structures, number of amino groups (n) and labels of the different amines.

Structure	Number of Amino Groups	Label
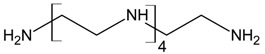	6	L6
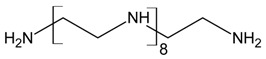	10	L10
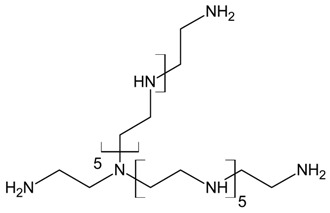	14	B14

**Table 2:** Sample labels and initial contacted amine concentration used in the clay modification experiments.

Sample (Label)	Amine Solution Initial Concentration (mM)	Initial Contacted Amine *C*_0_ (mmol/g_clay_)
L6-25	25	0.5
L6-70	70	1.5
L6-135	135	3.1
L6-175	175	4.0
L10-15	15	0.3
L10-25	25	0.6
L10-35	35	0.8
L10-60	60	1.4
L10-90	90	2.1
B14-15	15	0.3
B14-25	25	0.6
B14-40	40	0.9
B14-90	90	2.0

**Table 3:** Langmuir and Freundlich constants calculated from amines adsorption data on STx.

**Model**	***q*_max_(mmol/g)**	**K_L_(L/mmol)**	***R*^2^**
**Langmuir**	0.47	0.08	0.71
	**n**	**K_F_ (mmol/(g·(mmol/L)^1/n^)**	***R*^2^**
**Freundlich**	3.03	0.10	0.81

## Supplementary Materials

The following are available online at https://www.mdpi.com/2073-4360/11/5/897/s1, Figure S1. FT IR spectra of organo-clay L6-70 pure powder at increasing temperatures. Broken line: pure STx reference spectrum recorded at 100 °C. Arrows: bands assigned to L6 amine.
