# Supplementary material for: Polyamine-Based Organo-Clays for Polluted Water Treatment: Effect of Polyamine Structure and Content
Source: Polymers (Basel). 2019 May 16;11(5):897. doi: 10.3390/polym11050897 (PMC6571895; doi:10.3390/polym11050897)
Supplement: Supplementary file 1 [file polymers-11-00897-s001.pdf]

polymers

MDPI

Supplementary Materials: Polyamine-Based Organo-Clays for Polluted Water Treatment: Effect of Polyamine Structure and Content

Cinzia Cristiani, Elena Maria Iannicelli-Zubiani, Giovanni Dotelli, Elisabetta Finocchio, Paola Gallo Stampino and Maurizio Licchelli

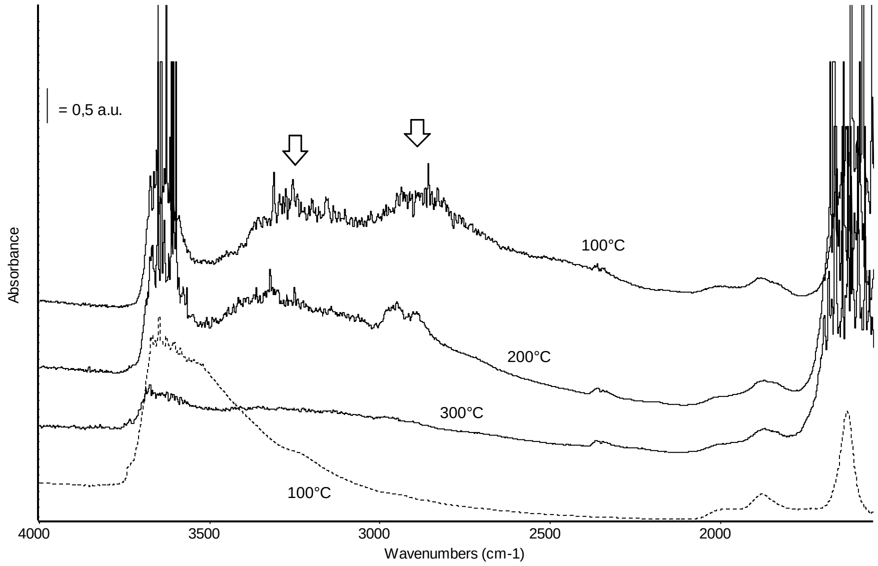

Figure S1. FT IR spectra of organo-clay L6-70 pure powder at increasing temperatures. Broken line: pure STx reference spectrum recorded at 100 °C. Arrows: bands assigned to L6 amine.
